# Evaluation of liposomal ciprofloxacin formulations in a murine model of anthrax

**DOI:** 10.1371/journal.pone.0228162

**Published:** 2020-01-24

**Authors:** Chad W. Stratilo, Scott Jager, Melissa Crichton, James D. Blanchard

**Affiliations:** 1 Bio Threat Defence Section, Suffield Research Centre, Defence Research and Development Canada, Ralston, Alberta, Canada; 2 Aradigm Corporation, Hayward, California, United States of America; CCAC, UNITED STATES

## Abstract

The *in vivo* efficacy of liposomal encapsulated ciprofloxacin in two formulations, lipoquin and apulmiq, were evaluated against the causative agent of anthrax, *Bacillus anthracis*. Liposomal encapsulated ciprofloxacin is attractive as a therapy since it allows for once daily dosing and achieves higher concentrations of the antibiotic at the site of initial mucosal entry but lower systemic drug concentrations. The *in vivo* efficacy of lipoquin and apulmiq delivered by intranasal instillation was studied at different doses and schedules in both a post exposure prophylaxis (PEP) therapy model and in a delayed treatment model of murine inhalational anthrax. In the mouse model of infection, the survival curves for all treatment cohorts differed significantly from the vehicle control. Ciprofloxacin, lipoquin and apulmiq provided a high level of protection (87–90%) after 7 days of therapy when administered within 24 hours of exposure. Reducing therapy to only three days still provided protection of 60–87%, if therapy was provided within 24 hours of exposure. If treatment was initiated 48 hours after exposure the survival rate was reduced to 46–65%. These studies suggest that lipoquin and apulmiq may be attractive therapies as PEP and as part of a treatment cocktail for *B*. *anthracis*.

## Introduction

*Bacillus anthracis*, the etiological agent of anthrax, is a spore-forming, gram-positive, rod shaped bacterium. Humans are usually infected by exposure to diseased animals or their products, although exposure from bioterrorism (USA, 2001) or accidental release (Sverdlovsk, USSR, 1979) has occurred. The route of exposure determines the disease caused: cutaneous, gastrointestinal or inhalation anthrax. Cutaneous anthrax is the most common form of the disease in humans, which has a mortality rate approaching 20%, due to septicemia, if untreated [1]. Inhalation anthrax has a mortality rate in humans of 45–80% even with antibiotic treatment at onset of symptoms [1–3].

Inhalational anthrax develops when *B*. *anthracis* spores are deposited in the alveolar spaces of the lung. The spores move, via a host carrier cell such as a macrophage or dendritic cell, from the alveolar spaces to the lymph nodes, where the spores germinate into vegetative bacteria [4–6]. Although, it has also been reported that spores can germinate in the lungs or within host cells and move to the lymph nodes without a carrier cell [7–9]. Once in the lymph node these bacteria replicate and produce exotoxins and a capsule, which results in bacterial escape from the lymph node to the bloodstream, disseminating throughout the body causing systemic disease [4, 8]. In humans, initial symptoms of respiratory anthrax are general flu like symptoms, lasting for two to three days. Sudden onset of acute illness is characterized by dyspnea, stridor, and fever leading to respiratory distress followed by death within days. Early initiation of treatment, including antimicrobials, is crucial for increased survivorship in animal models and humans [10–12].

Post exposure therapy protocols have been demonstrated in several animal models [13–15]. Most of these protocols include the use of a fluoroquinolone antibiotic, sometimes in combination with other antibiotics and/or post exposure vaccination [14, 15]. The inclusion of antibody based therapies has also been shown to improve survivorship in animal models [16, 17]. The Centre for Disease Control and Prevention (CDC) has provided guidance regarding post exposure prophylaxis (PEP) and treatment options for anthrax. PEP of an asymptomatic person includes antibiotic treatment using a fluoroquinolone antibiotic or doxycycline [18]. A cocktail of drugs, including a fluoroquinolone antibiotic and an antibiotic that inhibits protein synthesis, to supress anthrax toxin production, is recommended for treatment of *B*. *anthracis* infections. If meningitis is possible or confirmed, a β lactam antibiotic is included in the cocktail [18].

Administering antibiotics to target specific tissues, such as inhaled antibiotics that allow for delivering a relatively high concentration of drugs directly to lungs, would be an improvement compared to traditional systemic treatments. This approach would target the antibiotics to the lungs while plasma concentrations remain low, sparing the patient the potential side effects and toxicity associated with systemic administration of these drugs [19–22]. Aradigm corporation has developed a liposomal encapsulated ciprofloxacin for inhalation delivery. Two formulations have been developed; lipoquin containing only liposomal encapsulated ciprofloxacin and apulmiq, containing a mix of free and encapsulated ciprofloxacin; the development of these formulations have been reviewed [23]. Both drugs have been evaluated in human clinical trials [23–27]. Apulmiq completed phase 3 clinical trials for treatment of non-cystic fibrosis bronchiectasis patients with chronic *Pseudomonas aeruginosa* lung infections [28]. Once daily dosing of this product provides high sustained concentrations of ciprofloxacin to the lungs [29]. Liposome encapsulated drugs are ingested by phagocytic cells, including macrophages, and may accumulate in the tissues of the mononuclear phagocyte system, this may be of therapeutic value for some bacterial pathogens [30–32]. Lipoquin and apulmiq have specifically been shown to be phagocytized by macrophages and kill intracellular *Mycobacteria avium* and *M*. *abscessus* in *in vitro* and mouse lung infection models [33].

Liposomal ciprofloxacin formulations have been evaluated for several biothreat agents including *Francisella tularensis*, *Yersinia pestis*, and *Coxellia burnetii* [34–37]. All previous studies have demonstrated that these formulations are useful therapies for the biological agents of interest, both as a treatment and as a PEP [35–37]. This paper evaluates these two formulations of liposomal encapsulated ciprofloxacin as post exposure prophylaxis and as treatments in a mouse model of anthrax.

## Materials and methods

### Bacterial cultures and spores

The *B*. *anthracis* Ames strain is part of Defence Research and Development Canada—Suffield Research Centre (DRDC–SRC) permanent collection. It was originally isolated in 1981 from a dead cow in Texas. *B*. *anthracis* Ames spores were prepared according to the method of Leighton and Doi [38] with modification. The substantive changes to the protocol included: the sporulation media used was a casein hydrolysate yeast extract (CCY) liquid medium, vegetative cells were killed by incubation in 50% ethanol, and a density gradient centrifugation in Percoll (GE healthcare, USA) was used to clean the final spore prep [39]. Spores were confirmed by phase contrast microscopy and malachite green staining to be >99% dormant, bright-phase spores. This stock spore suspension was stored at 4°C and quantified by serial dilution enumeration after overnight incubation at 35°C on 5% sheep blood agar (SBA).

### Antibiotics

Ciprofloxacin (CIPRO, Bayer, USA), lipoquin (liposomal ciprofloxacin for inhalation) (Aradigm, USA) and apulmiq (dual release ciprofloxacin for inhalation) (Aradigm, USA) were used in this study. CIPRO (Bayer, USA) 20 mg ciprofloxacin base/mL and lipoquin 50 mg expressed as ciprofloxacin HCl/mL were used as provided. Apulmiq 35 mg, expressed as ciprofloxacin HCl/mL, was prepared by mixing equal volumes at a 50:50 ratio of lipoquin (50 mg ciprofloxacin HCL/mL) and CIPRO (20 mg ciprofloxacin HCL/mL).

### Animals

Pathogen-free female BALB/c mice (Charles River Laboratories, Quebec, Canada) weighing approximately 20 g were used throughout the study. Animals were sorted randomly into groups of five per cage. Animals were housed in Allentown NexGen EDGE cage system with Micro Barrier top with enrichment. Animals were allowed access to food and water *ad libitum* and housed in groups of five in 12 hour light–dark cycles.

### Ethics statement

All procedures were performed in accordance with protocols approved by the Defence Research and Development Canada Suffield Research Centre Institutional Animal Care and Use Committee, and met or exceeded the standards of the Canadian Council on Animal Care (CCAC).

### Intranasal infection in BALB/c mice

The 50% lethal dose of *B*. *anthracis* Ames spores (LD_50_) in BALB/c mice was 3.4 x 10^4^ colony-forming units (CFU) when challenged via intranasal installation, which is in agreement with other published work [40]. Mice (BALB/c) (n = 5/group) were anesthetized with isoflurane and challenged via the intranasal (i.n.) route with approximately 10 LD_50_ *B*. *anthracis* Ames spores. Mice receiving the challenge dose displayed no symptoms until approximately 48 hours post exposure. Mice could display limited symptoms (hunching, piloerection, orbital tightening) up to two days prior to acute symptoms including laboured irregular breathing and lethargy, which would indicate end state infection and the mice being euthanized [41, 42]. To verify final bacterial concentrations and installation doses, serial diluted and plated colonies were enumerated after overnight incubation at 35°C on 5% sheep blood agar (SBA).

### Antibiotic efficacy *in vivo*

Apulmiq (50 mg/kg or 10 mg/kg) or lipoquin (50 mg/kg or 10 mg/kg) were administered once daily to mice anesthetized with isoflurane via the intranasal (i.n.) route. Ciprofloxacin (30 mg/kg) was administered twice daily to mice via intraperitoneal (i.p) injection. Both treatments were compared to phosphate buffer saline (PBS) controls.

For study one, a post exposure prophylaxis (PEP) therapy model was used; in which groups of five infected mice (three replicates) were treated with a 10mg/kg or a 50 mg/kg dose at 24 hr post-challenge and continued for 7 days. In the second study, a treatment model was used; in which groups of five infected mice (three replicates) were treated 48 hr post-challenge (at the onset of the first symptoms of infection) with a 50 mg/kg of apulmiq or lipoquin and continued for 7 days. In the third study, a post exposure prophylaxis (PEP) therapy model was used; groups of five infected mice (three replicates) were provided therapy with a 50mg/kg dose at 24 hr post-challenge and continued for 3 days. In all studies, animals were evaluated post therapy for at least 30 days.

To assess bacterial burden control mice were culled as acute symptoms developed at which time livers, lungs, and spleens were harvested and processed to determine bacterial loads. Mice that survived to end of experiment were euthanized and livers, lungs, and spleens were excised, and the homogenates were plated on SBA to determine the bacteria load based on the presence of *B*. *anthracis*.

### Data analysis

For all experiments, Kaplan-Meyer survival curves were compared by the log-rank (Mantel-Cox) test using Prism Version 6.01, GraphPad Software. *In vivo* experiments were repeated three times using groups of five. Significance of lung CFU vs. control mice were calculated using Student’s t Test for unpaired data.

## Results

The *in vivo* efficacy of lipoquin and apulmiq delivered by intranasal instillation was studied at different doses and schedules in both a post exposure prophylaxis (PEP) therapy model (therapy initiated 24 hours after exposure) and in a delayed treatment model of murine inhalational anthrax (treatment initiated 48 hours after exposure, approximately at the onset of first symptoms in this model). Unencapsulated free ciprofloxacin delivered via the inhalation route has a very short half-life, approximately one hour, and is not an effective antimicrobial [43, 44]. It is also very irritating and not well tolerated by the animal models; therefore it was not included as a control in the experimental plan [37].

Mice in the ciprofloxacin treatment groups received ciprofloxacin at 30 mg/kg (b.i.d.) via the i.p. route of administration. This dosing regimen was selected to ensure that the plasma area under the curve (AUC) for a 24 hour period was similar in mice compared to the human label dose (11.6 μg hr/g compared to 10.7 +/- 2.6 μg hr/g) [37, 45]. Both lipoquin and apulmiq were administered at 50 mg/kg and 10 mg/kg doses for PEP and 50mg/kg for the treatment model. These doses were chosen for consistency between earlier studies, with the lower dose approximating a human dose based on plasma concentrations [35–37].

### Efficacy study 1

**Fig 1:**
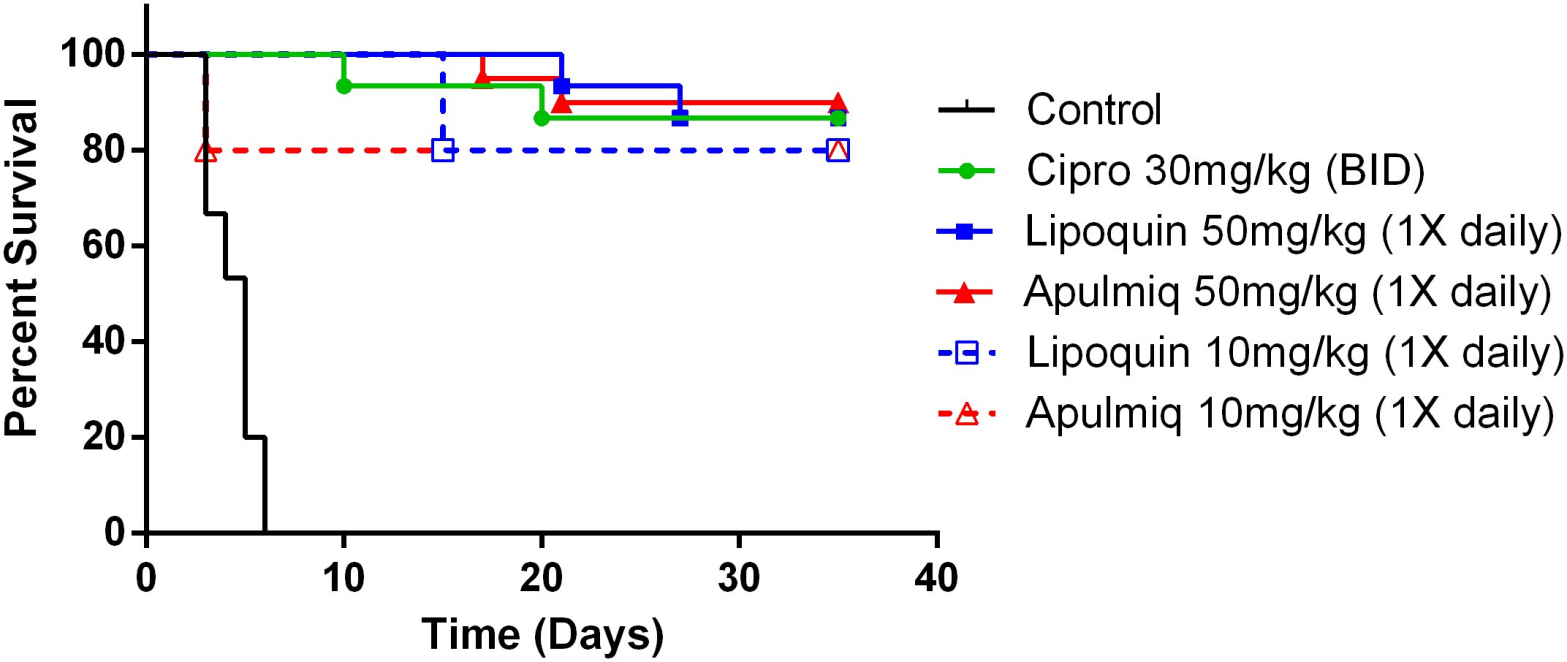
The efficacy of ciprofloxacin, lipoquin and apulmiq was evaluated in a post-exposure prophylaxis model after a lethal *B*. *anthracis* Ames challenge. Balb/c mice were challenged with 10 LD_50_ of *B*. *anthracis* Ames, and were treated as described, 24 hours after exposure for seven days. Therapeutic efficacy of intranasal delivered apulmiq and lipoquin (once daily) was compared at 50 mg/kg and 10 mg/kg versus intraperitoneal delivered ciprofloxacin (b.i.d.) and PBS-treated control mice. Graphs show the survival of mice following 7 days of therapy. All treatments improved survival compared to PBS (P < 0.001). There was no significant difference between the two concentrations of lipoquin and apulmiq administered.

The efficacy of ciprofloxacin, lipoquin and apulmiq was evaluated in a post-exposure prophylaxis model after a lethal *B*. *anthracis* Ames challenge. Mice were challenged with 10 LD_50_ of *B*. *anthracis* Ames, and were treated as described, 24 hours after exposure for seven days. Three replicates of the experiment occurred. The survival curves for the ciprofloxacin, lipoquin and apulmiq cohorts differed significantly (p<0.0001) from the vehicle control cohort (Fig 1). Median time to end state infection for control animals was 4 days post challenge, all control animals succumbed or reached end state infection by day six. Percent survival 30 days post therapy for each cohort was ciprofloxacin (87%), lipoquin (87%) and apulmiq (90%). Survival of mice that received a reduced daily dose of the lipoquin or apulmiq of 10 mg/kg for seven days was 80% for both formulations and not significantly different at the end of the experiment from mice that were dosed at 50 mg/kg for seven days (Fig 1).

### Efficacy study 2

**Fig 2:**
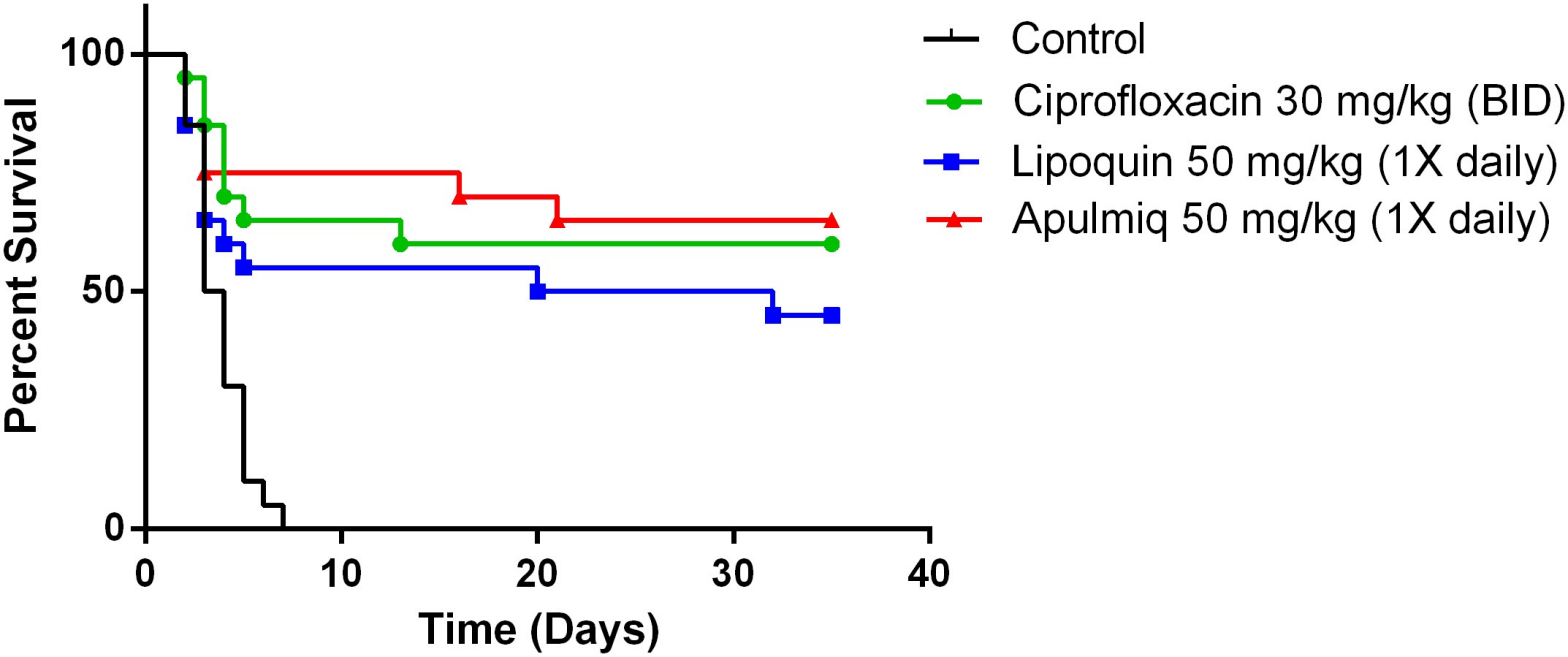
The efficacy of ciprofloxacin, lipoquin and apulmiq was evaluated in a treatment model after a lethal *B*. *anthracis* Ames challenge. Balb/c mice were challenged with 10 LD_50_ of *B*. *anthracis* Ames, and were treated as described, 48 hours after exposure for seven days. Efficacy of mice (n = 5x3) versus PBS-treated control mice was evaluated. Therapeutic efficacy of intraperitoneal delivered ciprofloxacin (b.i.d.) and intranasal delivered apulmiq and lipoquin (once daily) were compared. All treatments improved survival compared to PBS (P < 0.001).

The efficacy of ciprofloxacin, lipoquin and apulmiq as a treatment model after a lethal *B*. *anthracis* challenge was investigated. Mice were challenged with 10 LD_50_ of *B*. *anthracis* Ames and were dosed, as described, 48 hours after exposure for seven days. The survival curves for the ciprofloxacin, lipoquin and apulmiq cohorts differed significantly (p<0.0001) from the vehicle cohort (Fig 2). The median survival for the vehicle control cohort was 3 days with all control animals reaching end point by day 7. All three treatments provided increased survival, lipoquin (46%), apulmiq (65%) and ciprofloxacin (64%) until the end of the experiment compared to controls (p<0.0001). It should be noted that the majority of animals who succumb to infection did not survive until the end of treatment cycle (day nine).

### Efficacy study 3

**Fig 3:**
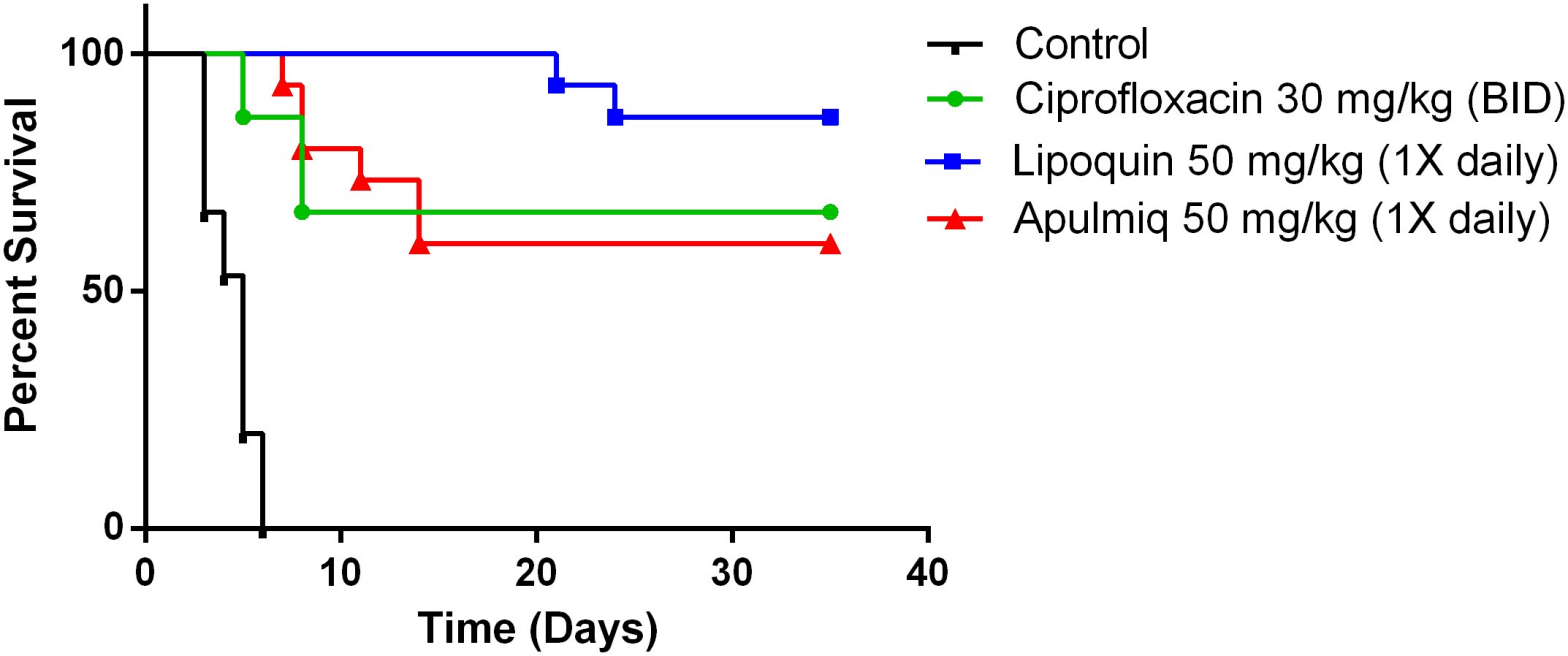
The efficacy of ciprofloxacin, lipoquin and apulmiq was evaluated in a post-exposure prophylaxis model after a lethal *B*. *anthracis* Ames challenge. Balb/c mice were challenged with 10 LD_50_ of *B*. *anthracis* Ames, and were treated as described, 24 hours after exposure for three days. Efficacy of mice (n = 5x3) versus PBS-treated control mice. Therapeutic efficacy of intraperitoneal delivered ciprofloxacin (b.i.d.) and intranasal delivered apulmiq and lipoquin (once daily) were compared. All treatments improved survival compared to PBS (P < 0.001).

To further evaluate the efficacy of liposomal antibiotics as a therapy for *B*. *anthracis* a dose sparing study was undertaken. Mice were challenged with 10 LD_50_ of *B*. *anthracis* Ames and were treated, as described, 24 hours after exposure for three days. The survival curves for the ciprofloxacin, apulmiq and lipoquin cohorts differed significantly (p<0.0001) from the vehicle cohort (Fig 3). All three therapies provided increased survival, lipoquin (87%), apulmiq (60%) and ciprofloxacin (67%) until the end of the experiment compared to controls (p<0.0001). The median survival for the vehicle control cohort was 4 days with all animals succumbing by day 6.

**Fig 4:**
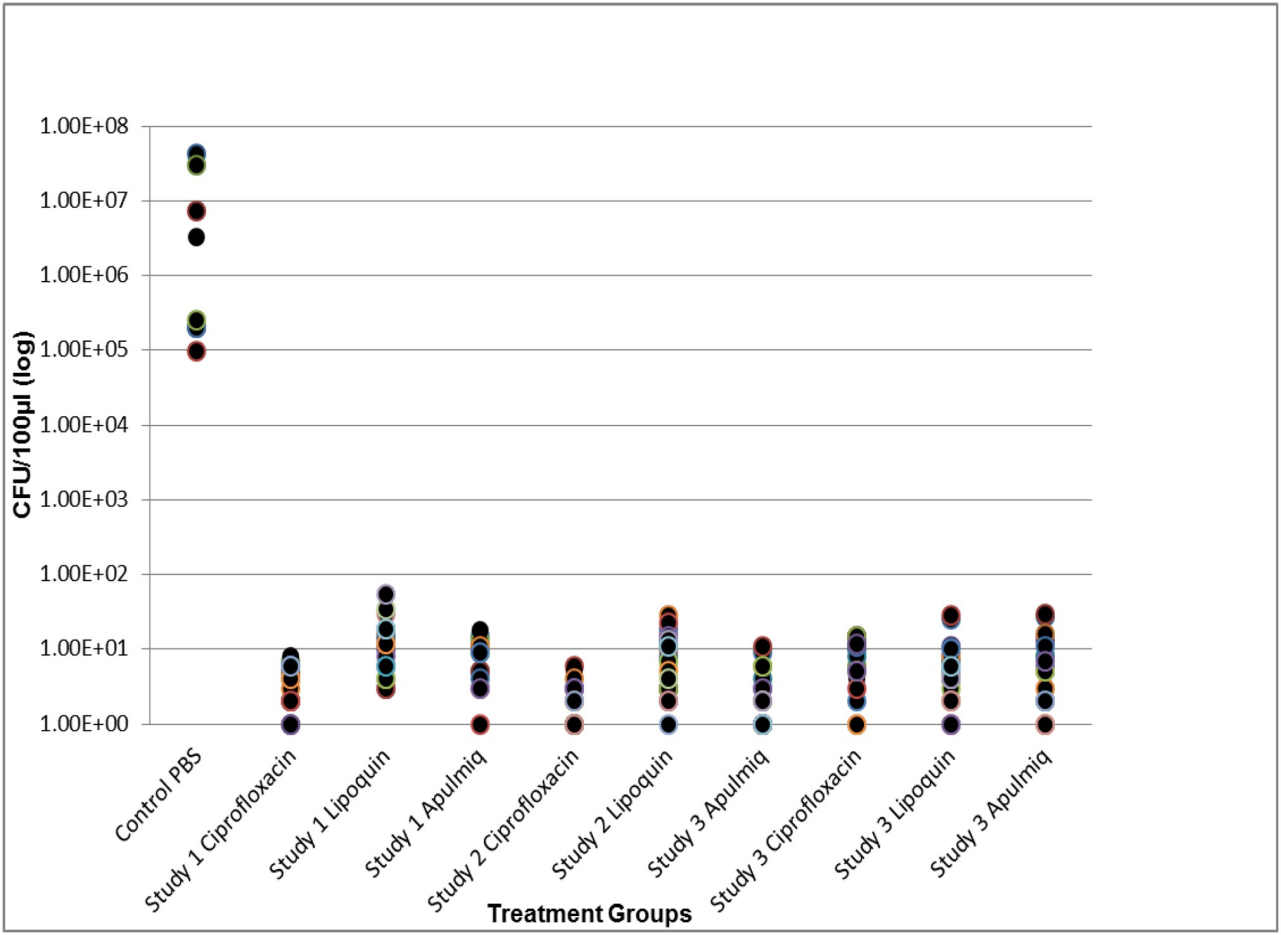
Bacterial load of lungs from surviving mice 30 days post therapy compared to lungs of treatment control mice (PBS) who succumb to infection. S1, S2, S3 refers to study number referenced in results. C is ciprofloxacin, A is apulmiq, and L is lipoquin. There was no significant difference in lung bacterial load between any treatment groups. All treatment groups were significantly different than PBS control group (p<0.001).

Upon study completion, spleens, livers, and lungs from surviving mice were excised and the homogenates were plated on SBA to determine the presence of *B*. *anthracis*. Consistent with this murine model of inhalational anthrax, residual *B*. *anthracis* were recovered from lungs of surviving mice (day 35 of experiment) that had received therapy. Although the bacterial counts were significantly less than lungs isolated from control animals (Fig 4). The bacterial loads of lungs isolated from mice from the various treatments group were not significantly different between groups. No bacteria were recovered from the spleens and livers of surviving mice that had received therapy. Control animals, which had succumbed to infection, had large numbers of bacteria isolated (mean numbers) from their liver (4.4 x10^7^ CFU), lungs (5.5 x10^7^ CFU) and spleen (2.8 x 10^7^ CFU). Positive lung results with negative spleens and livers are consistent with the infectious model post therapy [46].

## Discussion

Post exposure prophylaxis and treatment of anthrax includes antibiotic therapy, usually comprised of a fluoroquinolone antibiotic alone or as part of a cocktail. New formulations of ciprofloxacin, lipoquin and apulmiq, may be attractive alternatives to oral fluoroquinolones for treatment of anthrax, due to their dosing route, schedule, and attractive pharmacokinetics. Lipoquin and apulmiq are liposome encapsulated and administered via inhalation, and were developed for treating chronic lung infections and evaluated in human clinical trials [23, 26, 28]. Earlier work has demonstrated that the maximum concentration (Cmax) for an intranasal dose of lipoquin was approximately 100 fold higher in the lung and the AUC within the lungs for a 24 hour period for intranasal lipoquin was approximately 1000 fold higher compared to a dose of ciprofloxacin via the oral route [35]. Administration of the two drugs, lipoquin and apulmiq directly into the airway in mice provides a lung dose 20 fold higher than an i.p. dose of ciprofloxacin while offering a 10 fold reduction in systemic exposure [37]. Therefore, delivering antibiotics directly to the airway and lungs, enables higher antibiotic concentration at the site of infection or initial mucosal entry of the infectious agents as well as lower systemic exposure, reducing the chance of side effects, including microbiome impacts from therapy. Furthermore, providing an antibiotic formulation after a biological agent exposure may prevent the establishment of an infection and the appearance of symptoms since a large sustained targeted dose of antibiotic is provided directly to the site of initial exposure.

When mice were treated for seven days starting at 24 hr post-challenge, the mice treated with apulmiq, lipoquin or ciprofloxacin had 87–90% mean survival. In contrast, when therapy started at 24 hr after exposure and continued for 3 days, the lipoquin-treated groups had the highest mean survival rate (87%) compared to mean survival for apulmiq and ciprofloxacin (60% and 67% respectively). One possible explanation for lipoquin treatment having better efficacy shortly after exposure than apulmiq and free ciprofloxacin (i.p.) is that it has the highest resultant dose of ciprofloxacin in liposomal form, i.e., 50 mg/kg (100% of drug) vs. 35 mg/kg (71% of drug) for apulmiq and 0 mg/kg (0% of drug) for free ciprofloxacin. Encapsulated antibiotics are phagocytosed by macrophages therefore allowing the antibiotic to be in close proximity to the newly germinated *B*. *anthracis* vegetative cells in the macrophages and during the outgrowth of spores in the draining lymph nodes [7, 9].

When dosing of the encapsulated ciprofloxacin was reduced to 10 mg/kg for seven days the survival rates of the treated mice did not significantly change (80% for both formulations at 10 mg/kg vs 87% and 90% for lipoquin and apulmiq respectively at 50 mg/kg). When therapy was delayed until 48 hrs post challenge, the efficacy of the encapsulated formulations was reduced as did the efficacy of systemic treatment with traditional i.p. ciprofloxacin. At this time point lipoquin, apulmiq, and ciprofloxacin had 46–65% mean survival at end of the experiment. The relative efficacy of the antibiotics was not significantly different; apulmiq had the highest survival rate in both the groups that were treated for seven days. Earlier therapy (initiation at 24hrs vs 48hrs) did offer significantly improved survival rates for lipoquin (p<0.009), and were marginally improved for apulmiq (p<0.048) treatment groups but not for ciprofloxacin (p<0.069) treatment groups. This may be due to the development of a systemic infection; therefore targeted therapy to the site of initial infection would no longer be as effective.

Both apulmiq and lipoquin attain high efficacy (i.e., survival of 87–90%) against inhalation *B*. *anthracis* Ames infection in mice if therapy is initiated within 24 hours of exposure. If length of the treatment period was extended and vaccination was provided during the therapy period high survival would be likely [14]. The eradication of the spores that remain in the lungs or protective immunity would need to be acquired to ensure protection from residual spores.

In conclusion, inhaled encapsulated forms of ciprofloxacin, lipoquin and apulmiq, may be an attractive alternative to traditional oral fluoroquinolone antibiotics for PEP and treatment of pulmonary anthrax. Encapsulated ciprofloxacin has improved or equal efficacy as a PEP or treatment for anthrax in a mouse model and has a more attractive dosing schedule compared to oral ciprofloxacin in humans. Future studies that deliver these drugs in an aerosol form to additional animal models, including nonhuman primates, for prophylaxis and treatment of bacterial biothreat agent infections, should refine the efficacy of this drug as a general pre and post exposure prophylaxis and treatment for these agents of interest.

## Supporting information

S1 Table Survivor ship data from experiments. Days = number of days during experiment (Day 35 is end of experiment). 0 = survived, 1 = terminated. Groups of five mice per cage, replicate cages.
(PDF)

S1 File NC3Rs arrive guidelines checklist. (PDF)
