# Supplementary material for: Evaluation of liposomal ciprofloxacin formulations in a murine model of anthrax
Source: PLoS One. 2020 Jan 24;15(1):e0228162. doi: 10.1371/journal.pone.0228162 (PMC6980410; doi:10.1371/journal.pone.0228162)
Supplement: S1 Table — Days = number of days during experiment (Day 35 is end of experiment). 0 = survived, 1 = terminated. Groups of five mice per cage, replicate cages. (PDF) [file pone.0228162.s001.pdf]

Figure 1

24hours f	Days	Control	Cipro 30mg/kg	Lipoquin 50mg/k	Apulmiq 50mg/kg (1X daily)
M1	3	1			
M2	3	1			
M3	3	1			
M4	3	1			
M5	3	1			
M6	5	1			
M7	5	1			
M8	5	1			
M9	5	1			
M10	6	1			
M11	4	1			
M12	4	1			
M13	5	1			
M14	6	1			
M15	6	1			
Cipro M1	10		1		
Cipro M2	35		0		
Cipro M3	35		0		
Cipro M4	35		0		
Cipro M5	35		0		
Cipro M6	20		1		
Cipro M7	35		0		
Cipro M8	35		0		
Cipro M9	35		0		
Cipro M10	35		0		
Cipro M11	35		0		
Cipro M12	35		0		
Cipro M13	35		0		
Cipro M14	35		0		
Cipro M15	35		0		
Lipo M1	35			0	
Lipo M2	35			0	
Lipo M3	35			0	
Lipo M4	35			0	
Lipo M5	35			0	
Lipo M6	35			0	
Lipo M7	35			0	
Lipo M8	27			1	
Lipo M9	35			0	
Lipo M10	35			0	
Lipo M11	35			0	
Lipo M12	35			0	
Lipo M13	21			1	
Lipo M14	35			0	
Lipo M15	35			0	

Apulm M1	35				0
Apulm M2	35				0
Apulm M3	35				0
Apulm M4	35				0
Apulm M5	35				0
Apulm M6	21				1
Apulm M7	35				0
Apulm M8	35				0
Apulm M9	35				0
Apulm M1(	35				0
Apulm M1:	35				0
Apulm M1:	35				0
Apulm M1:	17				1
Apulm M1‹	35				0
Apulm M1!	35				0
Apulm M1(	35				0
Apulm M1:	35				0
Apulm M1!	35				0
Apulm M1!	35				0
Apulm M2(	35				

Figure 2

48hours 7 (	Days	Control	Cipro 30mg/kg	Lipoquin 50mg/kg	Apulmiq 50mg/kg (1X daily)
M1	3	1			
M2	3	1			
M3	4	1			
M4	5	1			
M5	7	1			
M6	2	1			
M7	2	1			
M8	2	1			
M9	3	1			
M10	4	1			
M11	3	1			
M12	3	1			
M13	4	1			
M14	5	1			
M15	5	1			
M16	3	1			
M17	3	1			
M18	4	1			
M19	5	1			
M20	6	1			
Cipro M1	35		0		
Cipro M2	2		1		
Cipro M3	4		1		
Cipro M4	35		0		

Cipro M5	35	0		
Cipro M6	4	1		
Cipro M7	5	1		
Cipro M8	35	0		
Cipro M9	35	0		
Cipro M10	35	0		
Cipro M11	35	0		
Cipro M12	35	0		
Cipro M13	35	0		
Cipro M14	3	1		
Cipro M15	3	1		
Cipro M16	4	1		
Cipro M17	35	0		
Cipro M18	13	1		
Cipro M19	35	0		
cipro M20	35	0		
Lipo M1	35		0	
Lipo M2	2		1	
Lipo M3	35		0	
Lipo M4	5		1	
Lipo M5	35		0	
Lipo M6	35		0	
Lipo M7	3		1	
Lipo M8	35		0	
Lipo M9	35		0	
Lipo M10	35		0	
Lipo M11	2		1	
Lipo M12	2		1	
Lipo M13	3		1	
Lipo M14	4		1	
Lipo M15	32		1	
Lipo M16	3		1	
Lipo M17	20		1	
Lipo M18	3		1	
Lipo M19	35		0	
Lipo M20	35		0	
Apulm M1	2			1
Apulm M2	35			0
Apulm M3	35			0
Apulm M4	35			0
Apulm M5	35			0
Apulm M6	35			0
Apulm M7	3			1
Apulm M8	35			0
Apulm M9	35			0
Apulm M1(	35			0
Apulm M1:	35			0

Apulm M12	3				1
Apulm M13	2				1
Apulm M14	35				0
Apulm M15	21				1
Apulm M16	35				0
Apulm M17	16				1
Apulm M18	35				0
Apulm M19	2				1
Apulm M20	35				0

Figure 3 Data

3days at 24	Days	Control	Cipro 30mg/kg	Lipoquin 50mg/kg	Apulmiq 50mg/kg (1X daily)
M1	3	1			
M2	3	1			
M3	3	1			
M4	3	1			
M5	3	1			
M6	5	1			
M7	5	1			
M8	5	1			
M9	5	1			
M10	6	1			
M11	4	1			
M12	4	1			
M13	5	1			
M14	6	1			
M15	6	1			
Cipro M1	35		0		
Cipro M2	5		1		
Cipro M3	5		1		
Cipro M4	8		1		
Cipro M5	35		0		
Cipro M6	35		0		
Cipro M7	35		0		
Cipro M8	35		0		
Cipro M9	8		1		
Cipro M10	35		0		
Cipro M11	35		0		
Cipro M12	35		0		
Cipro M13	35		0		
Cipro M14	35		0		
Cipro M15	8		1		
Lipo M1	35			0	
Lipo M2	21			1	
Lipo M3	35			0	
Lipo M4	35			0	

Lipo M5	35	0	
Lipo M6	35	0	
LIpo M7	24	1	
Lipo M8	35	0	
Lipo M9	35	0	
Lipo M10	35	0	
Lipo M11	35	0	
Lipo M12	35	0	
Lipo M13	35	0	
Lipo M14	35	0	
Lipo M15	35	0	
Apulm M1	35		0
Apulm M2	8		1
Apulm M3	7		1
Apulm M4	35		0
Apulm M5	35		0
Apulm M6	35		0
Apulm M7	14		1
Apulm M8	35		0
Apulm M9	35		0
Apulm M1[illegible]	35		0
Apulm M1[illegible]	8		1
Aplum M1[illegible]	35		0
Apulm M1[illegible]	11		1
Apulm M1[illegible]	35		0
Aplum M1[illegible]	14		1
